# Effect of Alpha-Lipoic Acid, Betaine, and L-Carnitine Supplementation on Gut Microbiota and Obesity Biomarkers in Mice

**DOI:** 10.3390/nu18060925

**Published:** 2026-03-14

**Authors:** Hye-Jin Kim, Jongbin Park, Soomin Oh, Dongwook Kim, Hee-Jin Kim, Cheorun Jo, Eun Bae Kim, Aera Jang

**Affiliations:** 1 Department of Applied Animal Science, College of Animal Life Science, Kangwon National University, Chuncheon 24341, Republic of Korea; cjsgusehd123@naver.com (H.-J.K.);; 2 Department of Agricultural Biotechnology, Center for Food and Bioconvergence, and Research Institute of Agriculture and Life Science, Seoul National University, Seoul 08826, Republic of Korea; 3 Poultry Research Institute, National Institute of Animal Science, Pyeongchang 25342, Republic of Korea

**Keywords:** obesity, gut microbiota, L-carnitine, 16S rRNA sequencing, correlation

## Abstract

**Background/Objectives:** This exploratory study (*n* = 6 per group) investigated the associations between supplementation with α-lipoic acid (AL), betaine (BT), and L-carnitine (LC) and gut microbiota composition in a high-fat diet (HFD)-induced obesity mouse model. **Methods:** Four-week-old male C57BL/6J mice were fed a control diet (10% fat), HFD (60% fat), or HFD supplemented with AL, BT, or LC (300 mg/kg BW/day) for nine weeks. **Results:** All three compounds were associated with shifts in microbial composition compared to the HFD-only group. While AL and BT supplementation moderately modulated specific Firmicutes and Bacteroidetes taxa, LC supplementation was linked to a more pronounced reduction in the Firmicutes/Bacteroidetes ratio and a decreased abundance of genera such as Christensenellaceae, Lachnospiraceae, and *Coprococcus 3*. These microbial changes were correlated with obesity-related metabolic and adiposity markers, including leptin and lipid parameters. Furthermore, functional profiling via PICRUSt suggested potential alterations in amino acid metabolism; however, these findings represent inferred metabolic potential rather than direct metagenomic measurements. **Conclusions:** Collectively, these results indicate differential associations between dietary supplementation and gut microbiota composition in HFD-fed mice. Although this study was conducted within an exploratory framework and utilized a modest sample size, the observed microbial shifts consistently paralleled metabolic alterations, supporting biologically plausible associations that warrant further mechanistic investigation.

## 1. Introduction

Obesity is a complex, multifactorial chronic metabolic disorder characterized by excessive fat accumulation that impairs metabolic health [1,2]. Although a positive energy balance contributes to weight gain, obesity is influenced by a combination of genetic, endocrine, environmental, and behavioral factors. According to the World Health Organization (WHO), in 2022, approximately 43.5% of adults were classified as overweight and 15.8% as obese based on body mass index (BMI) criteria [3]. However, BMI is primarily an epidemiological indicator used for population-level classification and does not fully capture adiposity-related metabolic dysfunction. Therefore, obesity is increasingly defined in terms of excessive fat accumulation and associated metabolic disturbances rather than BMI alone. In experimental models, high-fat diet (HFD) feeding induces adiposity, dyslipidemia, and metabolic alterations that mimic aspects of human obesity [4]. Obesity is associated with an increased risk of hypertension, cardiovascular disease, type 2 diabetes, and certain cancers [5].

The development of obesity and metabolic syndrome is also closely linked to alterations in gut microbiota composition [6,7]. The gut microbiota comprises trillions of microorganisms that contribute to energy homeostasis, nutrient metabolism, and immune regulation [8]. Dietary patterns strongly influence microbial composition and function, and dysbiosis has been implicated in metabolic dysfunction. Through modulation of intestinal permeability, inflammatory mediators, and host energy metabolism, gut microbiota may contribute to obesity-related metabolic disturbances. Therefore, microbiota modulation has emerged as a potential strategy for improving metabolic health.

Various strategies, including dietary control, exercise, and medication, have been used to regulate obesity [1]. Conventional pharmacological treatments carry a risk of various adverse effects, and dietary interventions based on naturally derived bioactive food compounds with relatively favorable safety profiles have emerged as alternative therapeutic strategies for the management of obesity and metabolic diseases [9]. α-Lipoic acid (AL), betaine (BT), and L-carnitine (LC) are metabolically distinct bioactive compounds involved in redox regulation, one-carbon metabolism, and mitochondrial fatty acid transport, respectively, and have been reported to improve metabolic parameters in obesity models [10]. AL acts as a cofactor for enzymes involved in energy metabolism and is relatively abundant in animal-derived foods such as red meat, liver, and heart, as well as in plant-derived foods including spinach, tomatoes, broccoli, and rice [11,12]. BT, which is widely distributed in animals, plants, and microorganisms, is a stable and non-toxic natural compound that is abundantly present in foods such as wheat bran, wheat germ, and spinach and has been reported to exhibit alleviating effects on hepatic lipid accumulation in animal models [13,14]. LC naturally occurs in mammalian muscle tissues and can be consumed through animal-derived foods; it regulates energy metabolism by facilitating the transport of long-chain fatty acids across the mitochondrial membrane [10,15]. In our previous study, the administration of AL, BT, and LC at 300 mg/kg BW for 9 weeks has been reported to significantly reduce the levels of serum leptin, serum triglyceride, serum total cholesterol, and hepatic lipid and the size of epididymal adipose tissues in HFD-induced obese mice [10]. However, the relationship between the anti-obesity effect of AL, BT, and LC supplementation and the modulation of gut microbiota in mice remains poorly understood.

Despite their shared metabolic benefits, AL, BT, and LC differ substantially in their biochemical functions. AL primarily acts as a redox-active cofactor involved in mitochondrial enzyme complexes and cellular antioxidant defense. BT functions as a methyl donor and osmolyte, participating in one-carbon metabolism and lipid homeostasis. LC is biochemically involved in mitochondrial fatty acid transport by facilitating the translocation of long-chain fatty acids across the mitochondrial membrane; however, the metabolic effects of supplementation may depend on physiological context and baseline carnitine status. These distinct metabolic roles suggest that, beyond their systemic effects on adiposity and lipid metabolism, each compound may exert differential influences on gut microbial ecology under high-fat dietary conditions. Recent evidence indicates that the gut microbiota plays a central role in host energy balance, lipid metabolism, and inflammatory regulation in obesity [8]. However, whether metabolically distinct dietary regulators such as AL, BT, and LC differentially modulate gut microbiota composition in HFD-induced obesity has not been systematically compared.

Therefore, we hypothesized that supplementation with AL, BT, or LC would be associated with distinct microbiota-associated responses in HFD-fed mice. The primary objective of this study was not to identify a single superior compound but to comparatively evaluate microbiota modulation patterns induced by these metabolically distinct supplements and to explore their associations with obesity-related metabolic and adiposity markers.

## 2. Materials and Methods

### 2.1. Animals and Experimental Design

Male C57BL/6J mice (4-week-old) were purchased from Samtaco (Seoul, Korea). Only male mice were used to reduce variability associated with estrous cycle-related hormonal fluctuations, which may influence metabolic and gut microbiota parameters [16]. The mice were acclimatized to the environment for 7 days. Mice were housed under controlled temperature (22 ± 2 °C) and humidity (50–60%) with a 12 h light/dark cycle. The sample size (*n* = 6 per group) was selected based on prior HFD-induced obesity studies evaluating metabolic and microbiota-associated outcomes [17]. Given the exploratory nature of this microbiome analysis, the results should be interpreted accordingly. Healthy mice were randomly allocated into five different feeding groups (*n* = 6/group): (1) commercial feed containing 10% crude fat (CON); (2) commercial HFD containing 60% crude fat; (3) HFD with AL at 300 mg/kg body weight (BW)/day (HFD-AL); (4) HFD with BT at 300 mg/kg BW/day (HFD-BT); (5) HFD with LC at 300 mg/kg BW/day (HFD-LC). The control diet (CON; D12450J, Research Diets, New Brunswick, NJ, USA) provided 10% of total energy from fat, 20% from protein, and 70% from carbohydrate, with an energy density of 3.85 kcal/g. The high-fat diet (HFD; D12492, Research Diets, New Brunswick, NJ, USA) provided 60% of total energy from fat, 20% from protein, and 20% from carbohydrate, with an energy density of 5.24 kcal/g. Percentages refer to the proportion of total caloric intake (kcal%). Random allocation was performed using a simple randomization procedure. Food and water were provided ad libitum. Supplementation (AL, BT, and LC, 300 mg/kg BW/day) was orally administered by gavage every day for 9 weeks at the same time. The body weights of mice treated with AL, BT, and LC reported in previous studies fell within the working weight range (0.011–0.034 kg) specified in the Food and Drug Administration guidance (Table S1) [10,18]. The selected dose (300 mg/kg BW/day) was based on our previous study demonstrating metabolic improvements in the same HFD model [10]. Accordingly, the human equivalent doses for AL, BT, and LC were all calculated to be 1.44 g/day for a 60 kg human [18]. Although body surface area-based scaling yields a higher numerical human equivalent dose, such interspecies conversion does not directly translate to clinical intake recommendations. All experimental procedures were approved by the Animal Care and Use Committee of Kangwon National University, Korea (KIACUC-13-0004, approved on 12 March 2013). No animals were excluded from the analysis, and potential confounders such as treatment order, measurement order, or animal/cage location were not specifically controlled. Blinding was not performed during allocation, experimental conduct, outcome assessment, or data analysis. No specific humane endpoints beyond standard institutional guidelines were predefined for this study. Animals were monitored daily for general health status and signs of distress, including reduced mobility, abnormal posture, piloerection, and rapid weight loss. Humane endpoints were defined according to institutional animal care guidelines, including severe weight loss (>20%), persistent lethargy, or signs of severe distress requiring early euthanasia. No animals met these criteria during the study.

### 2.2. Sample Collection and DNA Extraction

After 9 weeks of supplementation, a fecal sample was collected from each mouse (*n* = 6/group), and mice were anesthetized with diethyl ether, and blood was collected by cardiac puncture prior to sacrifice. The collected fecal sample was stored at −80 °C until analysis. The total bacterial genomic DNA from the fecal sample was extracted using a Maxwell^®^ RSC PureFood GMO and Authentication Kit (Promega, Madison, WI, USA) according to the manufacturer’s protocol. DNA samples were stored at −20 °C until analysis.

### 2.3. PCR Amplification of 16S Ribosomal RNA Gene (rRNA)

The V3–V4 region of the bacterial 16S rRNA gene was amplified from the extracted DNA using F319 and R806 primer sets [19]. The amplification of the DNA templates (12.5 ng/mL) was performed using a KAPA HiFi Hotstart PCR Kit (Kapa Biosystems, Wilmington, MA, USA). After PCR amplification, secondary amplification for attaching Illumina Nextera barcodes was performed using primers, including the i5 forward primer and i7 reverse primer. PCR products were purified using an Agencourt AMpure XP PCR Purification Kit (Beckman Coulter, Brea, CA, USA) following the manufacturer’s protocol. The concentration of purified DNA was verified using the QuantiFluor R ONE dsDNA System (Promega, Madison, WA, USA). The product size and quality were tested using Bioanalyzer 2100 (Agilent Technologies, Santa Clara, CA, USA). The pooled libraries were sequenced using an Illumina MiSeq instrument and a MiSeq v3 Reagent Kit (Illumina, San Diego, CA, USA).

### 2.4. Microbiome Bioinformatics and Statistical Analysis

Microbial community analysis was performed using Quantitative Insights into Microbial Ecology 2 (QIIME 2) version 2021.4 and the SILVA database. Taxonomic assignment was performed using the SILVA 132 database, which was the stable release compatible with the QIIME2 pipeline at the time of analysis [20]. Chimeric sequences and low sequencing quality reads (Phread < Q20) were filtered using the QIIME 2 plugin DADA2 with a denoise-paired option. Briefly, both forward and reverse reads were trimmed to 13 base pairs from the left (to remove low-quality reads) and subsequently truncated at 250 base pairs. The SILVA 132 classifier (99% identity) trained by QIIME 2 fit-classifier-naïve-Bayes was used for classification. The database version was retained in this revision to ensure full comparability with the initially processed datasets and to avoid introducing reprocessing-related variation. Accordingly, interpretations emphasize higher-rank and community-level patterns that are less sensitive to database version changes. The generated sequences were assigned to amplicon sequence variants (ASVs) using the reference SILVA database. Alpha diversity indices (Shannon, Chao1, and Simpson) and taxonomic relative abundances were compared among groups using the Kruskal–Wallis test, as microbiome relative abundance data did not meet normality assumptions and sample sizes were modest (*n* = 6 per group). When a significant overall group effect was detected, post hoc pairwise comparisons were conducted using Dunn’s multiple comparison test with Bonferroni correction to control false-positive findings arising from multiple testing. Beta diversity differences were evaluated using UniFrac distances and visualized via principal coordinate analysis (PCoA) using EMPeror software (version 2022.2). Phylogenetic investigation of communities by reconstruction of unobserved states (PICRUSt) was used to determine the metabolic function of the metagenomes obtained from the 16S rRNA gene using the Kyoto Encyclopedia of Genes and Genomes (KEGG) ortholog classification [21]. Functional pathway profiles were inferred using PICRUSt based on 16S rRNA gene data and represent predictive metabolic potential rather than direct metagenomic measurements. For functional prediction analysis, LEfSe was performed using the non-parametric Kruskal–Wallis test (α = 0.05) followed by linear discriminant analysis (LDA) with a threshold score > 2.0 [22]. Functional profiles inferred by PICRUSt represent predicted metabolic potential rather than direct metagenomic measurements.

To examine microbiota–host metabolic associations, Pearson’s correlation analyses were performed between taxonomic relative abundance and obesity-related metabolic markers. These markers—including serum leptin, serum triglycerides, total cholesterol, hepatic lipid content, and epididymal adipose tissue weight—were obtained from our previously published study conducted under identical experimental conditions using the same cohort of HFD-fed mice [10]. All parameters were measured as indicators of metabolic dysfunction and fat accumulation. Statistical analyses were conducted using SPSS (version 26.0; IBM, Armonk, NY, USA). A heatmap showing the correlation (r) between the relative abundance of the microbiome and biomarker was generated using Excel 2017 (Microsoft Corporation, Redmond, WA, USA).

## 3. Results

### 3.1. Microbiota Diversity

**Figure 1:**
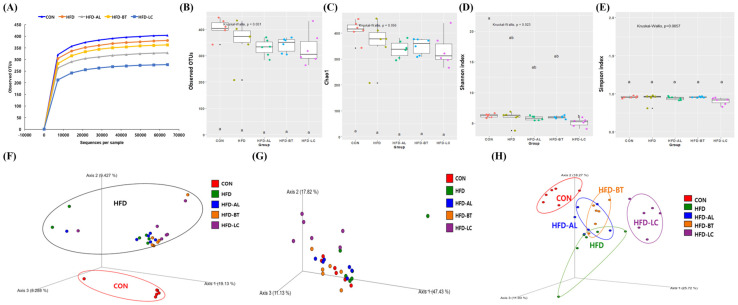
Alpha diversity and principal coordinate analysis (PCoA) of gut microbiota in mice. Rarefaction curves (**A**), observed OTUs (**B**), Chao1 (**C**), Shannon index (**D**), Simpson index (**E**), unweighted UniFrac distance (**F**), weighted UniFrac distance (**G**), and Bray–Curtis distance (**H**) of gut microbiota. ^a,b^ Different letters above the boxplots (**B**–**E**) indicate statistically significant differences among groups (*p* < 0.05). CON, control diet (10% fat); HFD, high-fat diet (60% fat); HFD-AL, HFD supplemented with α-lipoic acid (300 mg/kg BW/day); HFD-BT, HFD supplemented with betaine (300 mg/kg BW/day); HFD-LC, HFD supplemented with L-carnitine (300 mg/kg BW/day).

By analyzing bacterial 16S rRNA (V3–V4 regions) in fecal samples via pyrosequencing, we were able to determine the effects of AL, BT, and LC supplementation on the gut microbiota in HFD-fed mice. In total, 3,588,314 raw reads and 119,610 ± 27,903 reads per sample were obtained. Alpha diversity (observed OTUs, Chao1, Shannon, and Simpson indices) of the gut microbiota is shown in Figure 1. The Chao1 and Shannon values in the HFD group were lower than those in the CON group, but the difference was not statistically significant. Supplementation with AL, BT, and LC showed no significant differences in the observed OTUs, Chao1, and Simpson indices. However, the HFD-LC group showed significantly reduced Shannon values in relation to the CON group.

To evaluate the differences in gut microbial composition in mice, we performed principal coordinate analysis (PCoA) of fecal microbes (Figure 1F–H). The HFD-fed group (HFD, HFD + AL, HFD + BT, and HFD + LC) differed significantly from the CON group in the unweighted UniFrac distance results (Figure 1F). When weighted UniFrac distances were evaluated, each sample exhibited a significant degree of similarity (Figure 1G). HFD-fed groups were strongly distinguishable from the CON group in Bray–Curtis distance results, while the HFD-LC group exhibited significant differences from the HFD group (Figure 1H).

### 3.2. Composition of Gut Bacteria

**Figure 2:**
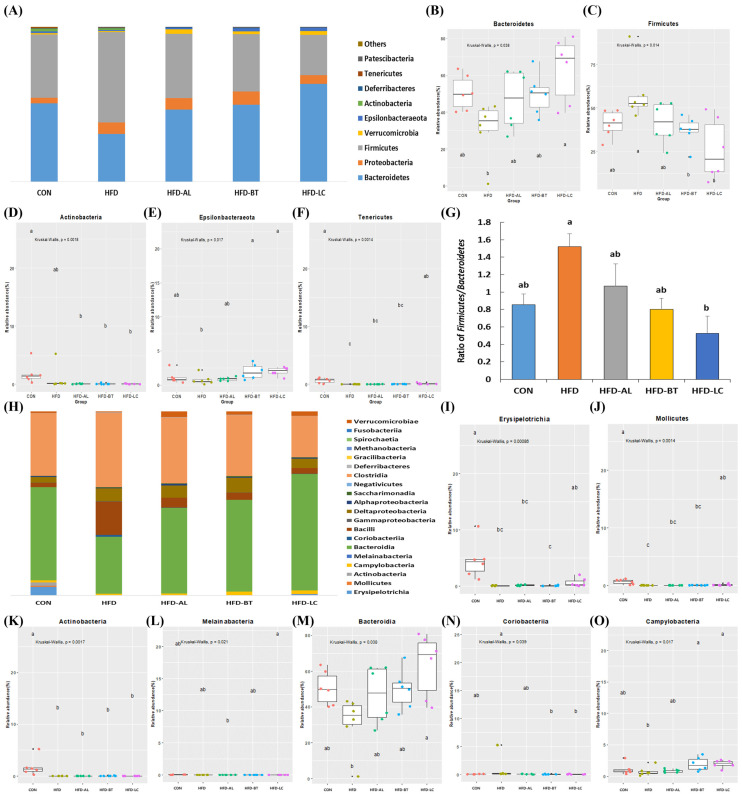
Relative abundance of gut microbes in mice at the phylum and class levels. Relative abundance (%) at the phylum level (**A**), significantly different phyla (**B**–**F**, *p* < 0.05), and the Firmicutes/Bacteroidetes ratio (**G**). Relative abundance (%) at the class level (**H**); significantly different classes (**I**–**O**, *p* < 0.05). ^a–c^ Different letters above the boxplots (**B**–**F**,**I**–**O**) indicate statistically significant differences among groups (*p* < 0.05). CON, control diet (10% fat); HFD, high-fat diet (60% fat); HFD-AL, HFD supplemented with α-lipoic acid (300 mg/kg BW/day); HFD-BT, HFD supplemented with betaine (300 mg/kg BW/day); HFD-LC, HFD supplemented with L-carnitine (300 mg/kg BW/day).

The structural composition of the gut microbiota at the phylum level is shown in Figure 2A–G. In this study, the intestinal microbes in each group were mainly composed of Bacteroidetes, Proteobacteria, and Firmicutes (Figure 2A). Among the treatments, significantly different phyla are shown in Figure 2B–F. The HFD group showed a decrease in Bacteroidetes (*p* > 0.05), Actinobacteria (*p* > 0.05), Epsilonbacteraeota (*p* > 0.05), and Tenericutes (*p* < 0.05), while Firmicutes increased (*p* > 0.05) in relation to the CON group. However, the HFD-LC group had significantly downregulated Firmicutes and upregulated Bacteroidetes in relation to the HFD group, which was similar to the CON group. The Firmicutes to Bacteroidetes (F/B) ratio was 1.52 in the HFD group, which was two-fold higher than that in the CON group (Figure 2G). However, HFD-LCs decreased the F/B ratio in relation to the HFD group (*p* < 0.05).

At the class level (Figure 2H–O), 20 major classes are shown in Figure 2H, and significantly different classes are shown in Figure 2I–O. The HFD group had lower levels of Erysipelotrichia, Mollicutes, Actinobacteria, and Bacteroidia than the CON group. The HFD-LC group contained more Mollicutes, Campylobacteria, and Bacteroidia than the HFD group (*p* < 0.05), which was similar to the CON group. However, Coriobacteriia was lower in the HFD-LC group than in the HFD group (*p* < 0.05).

At the genus level, 47 significantly different genera are shown in Table 1. In the Bacteroidetes phylum, *Muribaculaceae*, *Muribaculum*, and *Muribaculaceae* decreased in the HFD group (*p* < 0.05). *Bacteroides*, *Alloprevotella*, and *Parabacteroides* in the Bacteroidetes phylum appear to decrease in the HFD group, while the HFD-LC group showed an increase in relation to the HFD group (*p* < 0.05). *Rikenella* levels in the HFD group were higher than those in the CON group, while those in the HFD-LC group were lower (*p* < 0.05), which were similar to those in the CON group. In the Firmicutes phylum, the HFD group showed a significant increase in *Lactococcus*, Christensenellaceae, Lachnospiraceae, *Blautia*, *Coprococcus 3*, *Lachnoclostridium*, *Negativibacillus*, and Erysipelotrichaceae in relation to the CON group. However, the HFD-LC group showed a decrease in the abundance of Christensenellaceae, Lachnospiraceae, *Blautia*, *Coprococcus 3*, *Lachnoclostridium*, and Erysipelotrichaceae (*p* < 0.05), similar to the CON group. In the Epsilonbacteraeota phylum, *Helicobacter* showed decreasing trends in the HFD group; however, it increased in the HFD-LC group (*p* < 0.05).

**Figure 3:**
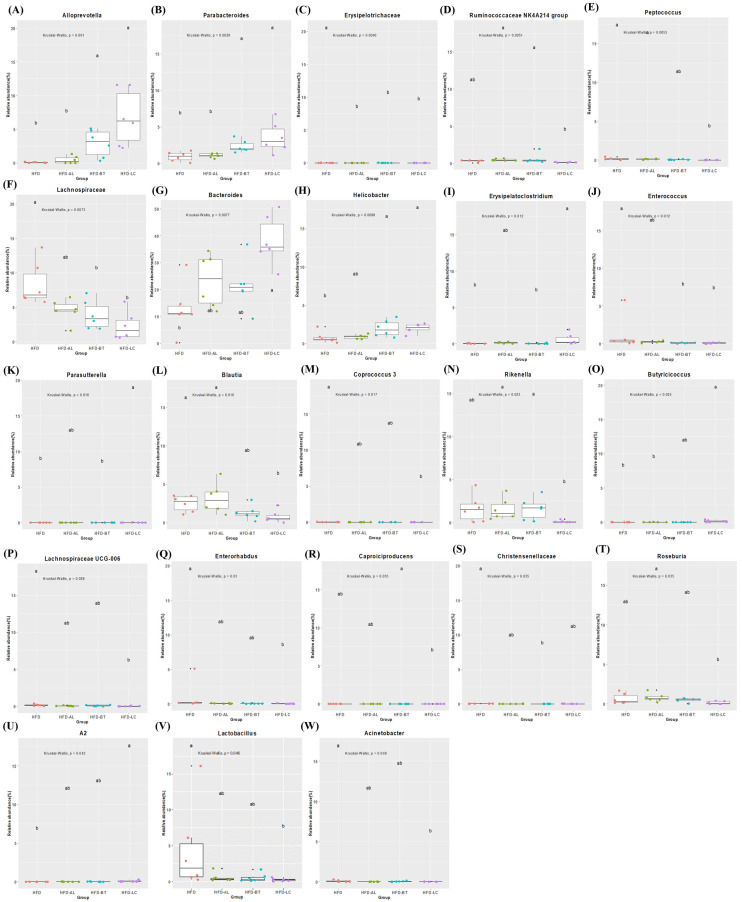
Differentially abundant taxa among HFD-treated groups (HFD, HFD-AL, HFD-BT, and HFD-LC). Each panel (**A**–**W**) represents the relative abundance (%) of a specific bacterial genus indicated at the top of each subplot. ^a,b^ Different letters above the boxplots (**A**–**W**) indicate statistically significant differences among groups (*p* < 0.05). HFD, high-fat diet (60% fat); HFD-AL, HFD supplemented with α-lipoic acid (300 mg/kg BW/day); HFD-BT, HFD supplemented with betaine (300 mg/kg BW/day); HFD-LC, HFD supplemented with L-carnitine (300 mg/kg BW/day).

Figure 3 compares the HFD and HFD-treatment groups. In this study, *Alloprevotella*, *Parabacteroides*, and *Helicobacter* in the HFD-BT and HFD-LC groups were higher than those in the HFD group (*p* < 0.05). Additionally, the HFD-LC group significantly increased *Bacteroides*, *Erysipelatoclostridium*, *Parasutterella*, and *Butyricicoccus* in relation to the HFD group. However, Erysipelotrichaceae decreased in the HFD-AL, HFD-BT, and HFD-LC groups, and Lachnospiraceae decreased in the HFD-BT and HFD-LC groups (*p* < 0.05). *Peptococcus*, *Blautia*, *Coprococcus 3*, Lachnospiraceae *UCG-006*, *Enterorhabdus*, *Lactobacillus*, and *Acinetobacter* were lower in the HFD-LC group than in the HFD group (*p* < 0.05).

### 3.3. Predictive KEGG Functional Profiling

**Figure 4:**
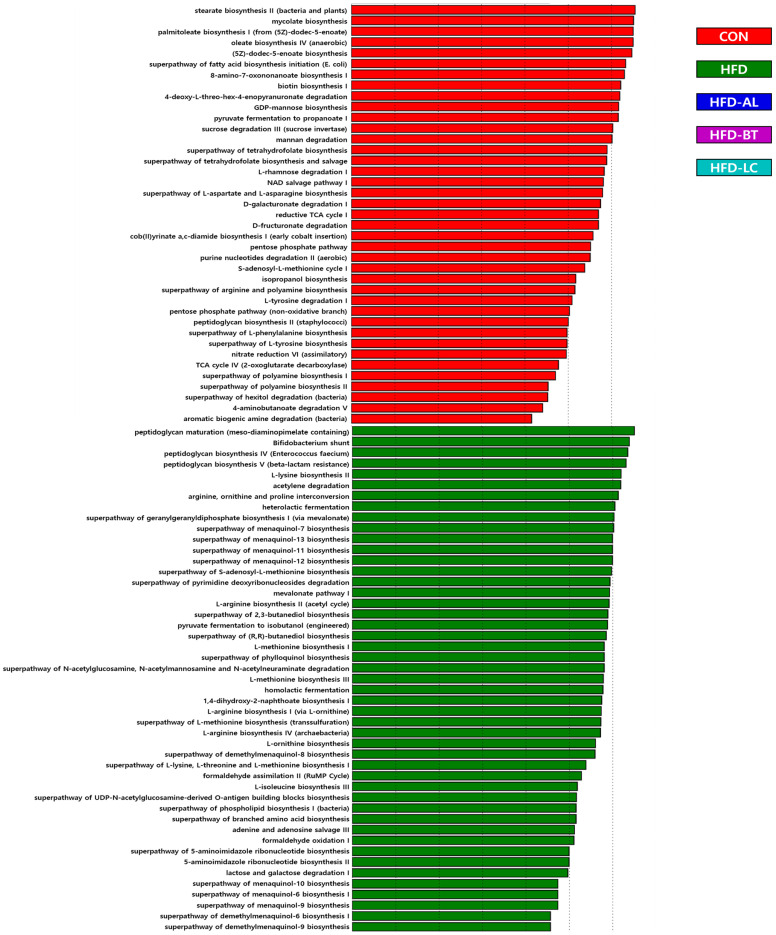

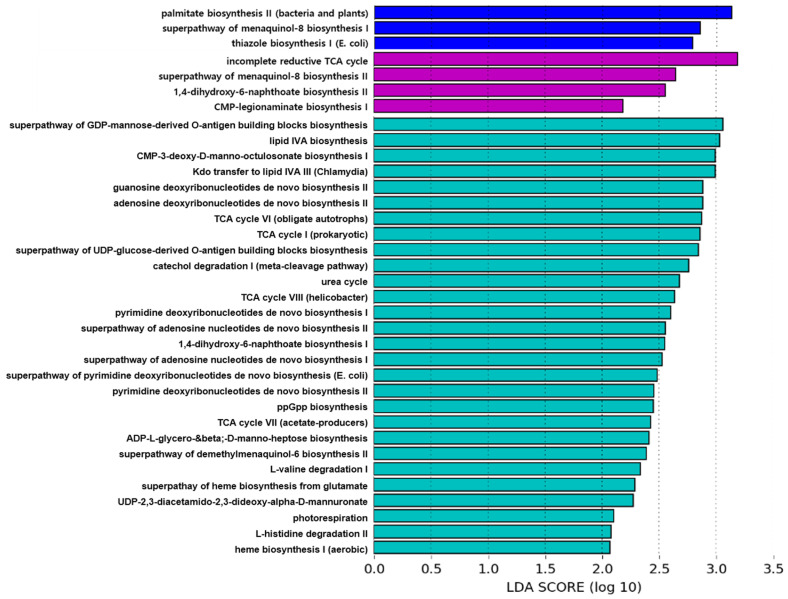
Predicted functional profiles of gut microbiota based on KEGG pathways. Functional capacity was inferred using PICRUSt and visualized by linear discriminant analysis (LDA) effect size (LEfSe). Functional predictions represent inferred metabolic potential derived from 16S rRNA gene data. CON, control diet (10% fat); HFD, high-fat diet (60% fat); HFD-AL, HFD supplemented with α-lipoic acid (300 mg/kg BW/day); HFD-BT, HFD supplemented with betaine (300 mg/kg BW/day); HFD-LC, HFD supplemented with L-carnitine (300 mg/kg BW/day).

KEGG categories of bacterial populations in the gut resulted in significant differences in 121 functional categories between the groups (Figure 4). A total of 39, 47, 3, 4, and 28 KEGG pathways were significantly upregulated in the CON, HFD, HFD-AL, HFD-BT, and HFD-LC groups, respectively. KEGG pathways involved in the TCA cycle, L-valine, and L-histidine degradation were enriched in the HFD-LC group. These results suggest that LC supplementation was associated with differences in predicted KEGG pathway profiles compared with HFD (Figure 4).

### 3.4. Correlation Between Obesity-Related Metabolic Markers and Gut Microbiota

**Figure 5:**
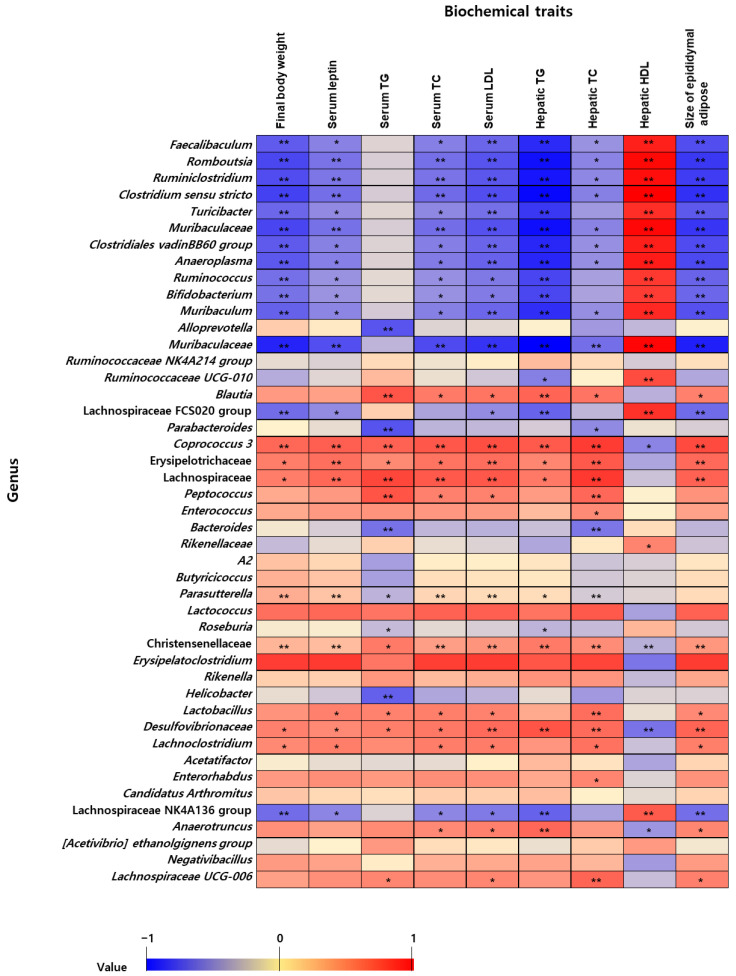
Heatmap of Pearson correlation coefficients between bacterial genera and metabolic/adiposity parameters. Columns represent obesity-related metabolic markers; rows represent bacterial genera. Color intensity indicates the correlation coefficient (r). Blue and red denote negative and positive correlations, respectively. * *p* < 0.05; ** *p* < 0.01.

To further evaluate the influence of gut microbiota on obesity in mice, the correlations between major biomarkers (Table S1) and significant genus results were analyzed (Figure 5) [10]. *Faecalibaculum*, *Romboutsia*, *Ruminiclostridium*, *Clostridium sensu stricto*, *Muribaculaceae*, *Clostridiales vadin BB60 group*, *Anaeroplasma*, *Ruminococcus*, *Bifidobacterium*, *Muribaculum*, and *Muribaculaceae* were negatively correlated with obesity-related metabolic markers such as final body weight, leptin, total cholesterol (TC), low-density lipoprotein (LDL) in serum, hepatic triglyceride (TG), and epididymal adipose (−0.823 ≤ r ≤ −0.374, *p* < 0.05). Serum TG levels were negatively correlated with *Alloprevotella* (r = −0.582, *p* < 0.01), Parabacteroides (r = −0.482, *p* < 0.01), and Bacteroides (r = −0.482, *p* < 0.01). However, *Coprococcus 3*, *Lactococcus*, and Christensenellaceae were positively correlated with obesity-related metabolic markers such as final body weight, levels of leptin, serum TC, hepatic TC, and TG, and the size of epididymal adipose tissues (0.440 ≤ r ≤ 0.678, *p* < 0.01). Additionally, increased Erysipelotrichaceae and Lachnospiraceae were correlated with increased leptin, serum LDL, and hepatic TC levels and epididymal adipose size (*p* < 0.01). Increased high-density lipoprotein (HDL) is associated with anti-obesity activity. In this study, *Faecalibaculum*, *Romboutsia*, *Ruminiclostridium*, *Clostridium sensu stricto*, *Muribaculaceae*, *Clostridiales vadin BB60 group*, *Anaeroplasma*, *Ruminococcus*, *Bifidobacterium*, *Muribaculum*, *Muribaculaceae*, *Ruminococcaceae UCG-010*, Lachnospiraceae *FCS020 group*, and Lachnospiraceae *NK4A136 group* were positively correlated with hepatic HDL (0.608 ≤ r ≤ 0.906, *p* < 0.01). HFD-LC reduced Christensenellaceae, Lachnospiraceae, and *Coprococcus 3*, which was positively linked with obesity indicators, including final body weight, leptin, serum TC, hepatic TC, and TG levels, as well as the size of epididymal adipose tissues (0.440 ≤ r ≤ 0.678, *p* < 0.01). However, *Bacteroides*, *Alloprevotella*, and *Helicobacter* were increased in the HFD-LC group, which was negatively correlated with serum TG (−0.582 ≤ r ≤ −0.482, *p* < 0.01). Several genera showed significant correlations with metabolic/adiposity parameters (Figure 5).

## 4. Discussion

In the present study, 16S rRNA gene sequencing was used to investigate the effects of AL, BT, and LC supplementation on gut microbiota composition in HFD-fed mice. HFD feeding was associated with a tendency toward reduced alpha diversity compared with the control diet, consistent with previous reports indicating that obesogenic diets may decrease microbial diversity in mice [5,8,23]. Reduced alpha diversity has often been linked to metabolic dysfunction; however, recent studies suggest that diversity metrics alone do not necessarily reflect functional or metabolic health status [24,25]. Indeed, dietary interventions such as plant-derived extracts or polyphenols have been shown to modulate or even reduce microbial diversity without uniform metabolic consequences [25,26]. In the present study, AL and BT supplementation did not significantly alter alpha diversity indices, whereas LC supplementation was associated with reduced Shannon diversity relative to CON. Although reduced alpha diversity is often associated with metabolic dysfunction, diversity alone does not necessarily reflect functional health status, and specific compositional shifts may be more relevant than overall richness [27,28]. Therefore, the biological implication of reduced diversity in the LC group warrants cautious interpretation.

Beta diversity analysis demonstrated clear separation between HFD-fed groups and the control diet, indicating that high-fat feeding substantially reshaped the overall microbial community structure. Among the supplementation groups, LC exhibited comparatively greater separation from HFD in Bray–Curtis distance, suggesting compound-specific modulation of community composition. Nevertheless, AL and BT also contributed to distinct clustering patterns relative to HFD, supporting the concept that metabolically different supplements may exert differential effects on gut microbial ecology [29,30].

At the phylum level, Firmicutes and Bacteroidetes dominated the murine gut microbiota, consistent with prior reports [28,31]. HFD feeding tended to increase Firmicutes and decrease Bacteroidetes, a pattern widely reported in HFD models [24,28]. LC supplementation was associated with partial restoration of this pattern, reflected in a lower Firmicutes/Bacteroidetes (F/B) ratio relative to HFD. Although earlier studies proposed the F/B ratio as a hallmark of obesity, its biological relevance remains debated across models and populations [25,32,33,34,35]. Therefore, F/B alterations observed here should be interpreted as part of broader compositional shifts rather than as a standalone biomarker.

At finer taxonomic levels, supplementation-specific patterns became more apparent. At the class level, LC supplementation was associated with increased *Mollicutes*, *Campylobacteria*, and *Bacteroidia* relative to HFD, while *Coriobacteriia* decreased. Similar modulation of HFD-induced class-level alterations has been reported with bioactive dietary compounds, including ginger and omega-3 fatty acids [8,36], suggesting that metabolically active supplements may partially counteract obesogenic microbiota shifts.

At the genus level, HFD feeding increased several taxa previously linked to diet-induced obesity, including members of Lachnospiraceae and genera such as *Blautia and Coprococcus* [32,34,35]. LC supplementation was associated with a reduced abundance of Christensenellaceae-, Lachnospiraceae-, and *Coprococcus 3*-related taxa and an increased abundance of *Bacteroides* and *Alloprevotella* compared with HFD. Enterotype-associated genera such as *Bacteroides*, *Ruminococcus*, and *Prevotella* have been described as diet-responsive microbial signatures [33], and shifts in these genera may reflect alterations in macronutrient metabolism rather than direct metabolic outcomes. BT supplementation also reduced Lachnospiraceae abundance, consistent with prior reports linking BT to modulation of gut barrier function and microbial composition [37]. Similarly, AL and BT groups demonstrated reductions in Erysipelotrichaceae relative to HFD, a family previously associated with diet-induced metabolic disturbance [32].

Regarding *Helicobacter*, abundance increased in the LC group relative to the HFD group. However, interpretations should remain cautious because this genus includes species that may act as pathobionts in murine models. Therefore, genus-level increases should not be universally interpreted as beneficial and require species-level resolution for biological clarification.

Predictive functional profiling revealed significant differences in KEGG pathway categories across dietary groups. HFD feeding was associated with enrichment of amino acid biosynthesis pathways, including branched-chain amino acid metabolism, consistent with previous obesogenic dietary models [38]. LC supplementation was associated with relative enrichment of pathways related to central carbon metabolism and amino acid degradation. Comparable modulation of microbial functional categories under HFD conditions has been reported with polyphenol, EGCG, or ginger supplementation [8,39]. Nevertheless, because these profiles were inferred using PICRUSt from 16S rRNA gene data, they represent predicted metabolic potential rather than direct metagenomic measurement and should therefore be considered hypothesis-generating.

Correlation analyses demonstrated associations between specific genera and metabolic/adiposity-related parameters. Several taxa were negatively correlated with body weight, leptin, lipid profiles, and epididymal adipose tissue weight, whereas others, including *Coprococcus 3* and Christensenellaceae-related taxa, showed positive correlations. Notably, genera enriched in the LC group, such as *Bacteroides* and *Alloprevotella*, were negatively correlated with serum triglyceride levels. However, the relationship between gut microbiota and obesity is bidirectional and shaped by host metabolism, dietary exposure, and microbial interactions. Accordingly, these correlations should not be interpreted as evidence of causal mechanisms linking supplementation to metabolic improvement.

Despite the strengths of the present study, several limitations should be considered when interpreting the findings. First, this study was conducted in a diet-induced obesity model using male C57BL/6J mice with a modest sample size (*n* = 6 per group). Although this model is widely used and responsive to high-fat feeding, it does not fully recapitulate the complexity of human obesity. Species-specific differences in gut microbiota composition, host metabolism, and immune regulation may limit direct extrapolation to humans. In addition, blinding was not implemented during experimental procedures or data analysis. Second, microbiota profiling was performed using 16S rRNA gene sequencing, and functional pathway analysis was inferred using PICRUSt. Therefore, functional predictions represent inferred metabolic potential rather than direct metagenomic measurement. In addition, relative abundance data are compositional in nature, and shifts in specific taxa may reflect proportional rather than absolute changes in microbial load. Third, taxonomic assignment was conducted using the SILVA 132 database; although consistent with the original analytical workflow, taxonomic resolution—particularly at the genus level—may vary across database versions. Finally, the supplementation dose (300 mg/kg BW/day) was selected based on prior murine studies. Although a human equivalent dose was estimated, differences in bioavailability, long-term safety, and lifestyle factors may influence translational applicability. Accordingly, the observed associations between microbiota modulation and metabolic/adiposity markers should be interpreted within the context of this experimental and exploratory framework.

## 5. Conclusions

Supplementation with AL, BT, and LC was associated with distinct alterations in gut microbiota composition in HFD-fed mice. Our study showed that LC supplementation was associated with comparatively greater shifts in specific microbial taxa and community structure. The HFD-LC group demonstrated a decreased F/B ratio and altered microbial composition. The observed decreases in Christensenellaceae, Lachnospiraceae, and *Coprococcus 3*, along with increases in *Bacteroides*, *Alloprevotella*, and *Helicobacter*, were correlated with phenotype indices related to metabolic and adiposity-related parameters. Among the three compounds, LC showed more pronounced microbiota-associated shifts that paralleled changes in metabolic and adiposity-related parameters. However, these findings reflect associative relationships within an experimental model and do not establish causal mechanisms. Overall, this study highlights differential microbiota responses to metabolically distinct dietary supplements and provides a foundation for future mechanistic and translational investigations.

## Figures and Tables

**Table 1:** Significant relative abundance (%) of gut microbes in mice at the genus level.

Trait	CON	HFD	HFD-AL	HFD-BT	HFD-LC
Bacteroidetes					
*Bacteroides*	22.80 ± 8.95 ^ab^	12.86 ± 9.41 ^b^	23.36 ± 9.88 ^ab^	21.53 ± 8.88 ^ab^	38.25 ± 9.13 ^a^
*Muribaculaceae*	2.93 ± 0.27 ^a^	0.57 ± 0.32 ^b^	0.68 ± 0.20 ^ab^	1.01 ± 0.3 ^ab^	0.56 ± 0.42 ^b^
*Muribaculum*	0.10 ± 0.08 ^a^	0.00 ± 0.00 ^b^	0.00 ± 0.00 ^b^	0.00 ± 0.00 ^b^	0.01 ± 0.02 ^b^
*Muribaculaceae*	8.53 ± 1.2 ^a^	4.17 ± 2.12 ^b^	5.67 ± 1.76 ^ab^	6.28 ± 2.05 ^ab^	3.79 ± 1.58 ^b^
*Alloprevotella*	0.54 ± 0.70 ^bc^	0.09 ± 0.08 ^c^	0.45 ± 0.56 ^c^	2.9 ± 2.04 ^ab^	6.74 ± 4.15 ^a^
*Rikenellaceae*	0.72 ± 0.67 ^a^	0.41 ± 0.45 ^ab^	0.19 ± 0.36 ^b^	0.25 ± 0.26 ^ab^	0.05 ± 0.06 ^b^
*Rikenella*	0.58 ± 0.34 ^ab^	1.64 ± 1.59 ^a^	1.54 ± 1.26 ^a^	1.60 ± 1.28 ^a^	0.12 ± 0.14 ^b^
*Parabacteroides*	1.64 ± 0.84 ^ab^	0.92 ± 0.64 ^b^	1.06 ± 0.29 ^ab^	2.32 ± 0.83 ^a^	3.53 ± 2.07 ^a^
Firmicutes					
*Enterococcus*	0.42 ± 0.30 ^a^	1.23 ± 2.24 ^a^	0.26 ± 0.12 ^ab^	0.11 ± 0.05 ^b^	0.10 ± 0.08 ^b^
*Lactobacillus*	1.20 ± 0.68 ^a^	4.48 ± 6.10 ^a^	0.58 ± 0.63 ^ab^	0.55 ± 0.61 ^ab^	0.26 ± 0.23 ^b^
*Lactococcus*	0.65 ± 0.25 ^b^	12.29 ± 13.84 ^a^	4.50 ± 3.01 ^ab^	3.01 ± 1.90 ^ab^	2.69 ± 2.98 ^ab^
Christensenellaceae	0.00 ± 0.00 ^b^	0.04 ± 0.01 ^a^	0.01 ± 0.01 ^ab^	0.01 ± 0.01 ^ab^	0.02 ± 0.02 ^b^
*Candidatus Arthromitus*	0.00 ± 0.00 ^b^	0.01 ± 0.01 ^ab^	0.04 ± 0.03 ^a^	0.01 ± 0.00 ^ab^	0.03 ± 0.07 ^ab^
*Clostridium sensu stricto*	2.96 ± 1.00 ^a^	0.01 ± 0.02 ^b^	0.00 ± 0.00 ^b^	0.00 ± 0.00 ^b^	0.00 ± 0.00 ^b^
*Clostridiales vadinBB60 group*	0.22 ± 0.22 ^a^	0.00 ± 0.00 ^b^	0.00 ± 0.00 ^b^	0.00 ± 0.00 ^b^	0.01 ± 0.01 ^b^
Lachnospiraceae	3.83 ± 0.88 ^b^	8.36 ± 3.15 ^a^	4.55 ± 1.64 ^ab^	3.88 ± 2.07 ^b^	2.30 ± 2.05 ^b^
*[Acetivibrio] ethanolgignens group*	0.16 ± 0.09 ^a^	0.13 ± 0.14 ^ab^	0.14 ± 0.11 ^ab^	0.06 ± 0.08 ^ab^	0.03 ± 0.06 ^b^
*A2*	0.00 ± 0.00 ^b^	0.00 ± 0.00 ^b^	0.01 ± 0.02 ^ab^	0.02 ± 0.03 ^ab^	0.10 ± 0.13 ^a^
*Acetatifactor*	0.08 ± 0.05 ^b^	0.35 ± 0.3 ^ab^	0.35 ± 0.30 ^ab^	1.10 ± 1.22 ^a^	0.16 ± 0.2 ^ab^
*Blautia*	0.67 ± 0.24 ^b^	2.51 ± 1.05 ^a^	3.19 ± 1.94 ^a^	1.32 ± 0.93 ^ab^	0.80 ± 0.80 ^b^
*Coprococcus 3*	0.00 ± 0.00 ^b^	0.02 ± 0.02 ^a^	0.01 ± 0.00 ^ab^	0.01 ± 0.01 ^ab^	0.00 ± 0.00 ^b^
*Lachnoclostridium*	0.47 ± 0.24 ^b^	3.39 ± 4.96 ^a^	0.8 ± 0.34 ^ab^	0.73 ± 0.41 ^ab^	0.61 ± 0.77 ^b^
Lachnospiraceae *FCS020 group*	0.09 ± 0.04 ^a^	0.02 ± 0.02 ^b^	0.05 ± 0.05 ^ab^	0.01 ± 0.01 ^b^	0.00 ± 0.01 ^b^
Lachnospiraceae *NK4A136 group*	7.5 ± 3.31 ^a^	3.03 ± 1.51 ^b^	3.66 ± 3.62 ^ab^	4.21 ± 1.33 ^ab^	2.95 ± 1.51 ^ab^
Lachnospiraceae *UCG-006*	0.07 ± 0.06 ^ab^	0.18 ± 0.11 ^a^	0.07 ± 0.04 ^ab^	0.11 ± 0.09 ^ab^	0.03 ± 0.04 ^ab^
*Roseburia*	0.17 ± 0.16 ^b^	0.65 ± 0.66 ^ab^	0.81 ± 0.53 ^a^	0.5 ± 0.24 ^ab^	0.16 ± 0.18 ^b^
*Peptococcus*	0.09 ± 0.08 ^ab^	0.18 ± 0.14 ^a^	0.13 ± 0.06 ^a^	0.06 ± 0.04 ^ab^	0.02 ± 0.02 ^b^
*Romboutsia*	0.72 ± 0.32 ^a^	0.00 ± 0.00 ^b^	0.00 ± 0.00 ^b^	0.00 ± 0.00 ^b^	0.00 ± 0.00 ^b^
*Anaerotruncus*	0.13 ± 0.03 ^b^	0.43 ± 0.29 ^ab^	0.58 ± 0.28 ^a^	0.30 ± 0.20 ^ab^	0.34 ± 0.18 ^ab^
*Butyricicoccus*	0.01 ± 0.01 ^b^	0.01 ± 0.01 ^b^	0.01 ± 0.02 ^ab^	0.01 ± 0.01 ^ab^	0.16 ± 0.16 ^a^
*Negativibacillus*	0.00 ± 0.00 ^b^	0.02 ± 0.02 ^a^	0.01 ± 0.01 ^ab^	0.02 ± 0.04 ^ab^	0.02 ± 0.02 ^ab^
*Ruminiclostridium*	0.04 ± 0.02 ^a^	0.00 ± 0.00 ^b^	0.00 ± 0.00 ^b^	0.00 ± 0.00 ^b^	0.00 ± 0.00 ^b^
*Ruminococcaceae NK4A214 group*	0.22 ± 0.07 ^bc^	0.31 ± 0.13 ^ab^	0.47 ± 0.13 ^a^	0.60 ± 0.66 ^ab^	0.12 ± 0.06 ^c^
*Ruminococcaceae UCG-010*	0.47 ± 0.26 ^a^	0.27 ± 0.14 ^ab^	0.21 ± 0.06 ^b^	0.11 ± 0.09 ^b^	0.12 ± 0.08 ^b^
*Ruminococcus*	2.07 ± 2.00 ^a^	0.00 ± 0.00 ^b^	0.00 ± 0.00 ^b^	0.00 ± 0.00 ^b^	0.00 ± 0.00 ^b^
Erysipelotrichaceae	0.01 ± 0.01 ^a^	0.00 ± 0.00 ^b^	0.00 ± 0.00 ^b^	0.00 ± 0.00 ^b^	0.01 ± 0.03 ^ab^
*Erysipelatoclostridium*	0.08 ± 0.07 ^ab^	0.05 ± 0.05 ^b^	0.16 ± 0.10 ^ab^	0.05 ± 0.07 ^b^	0.61 ± 0.76 ^a^
*Faecalibaculum*	1.95 ± 1.37 ^a^	0.00 ± 0.00 ^b^	0.00 ± 0.00 ^b^	0.00 ± 0.00 ^b^	0.00 ± 0.00 ^b^
*Turicibacter*	2.54 ± 2.13 ^a^	0.00 ± 0.00 ^b^	0.00 ± 0.00 ^b^	0.00 ± 0.00 ^b^	0.00 ± 0.00 ^b^
Erysipelotrichaceae	0.00 ± 0.00 ^b^	0.02 ± 0.01 ^a^	0.00 ± 0.01 ^b^	0.01 ± 0.02 ^b^	0.00 ± 0.00 ^b^
Actinobacteria					
*Bifidobacterium*	1.77 ± 1.77 ^a^	0.00 ± 0.00 ^b^	0.00 ± 0.00 ^b^	0.00 ± 0.00 ^b^	0.00 ± 0.00 ^b^
*Enterorhabdus*	0.06 ± 0.02 ^ab^	0.97 ± 2.02 ^a^	0.07 ± 0.05 ^ab^	0.06 ± 0.05 ^b^	0.05 ± 0.04 ^b^
Epsilonbacteraeota					
*Helicobacter*	1.14 ± 0.92 ^ab^	0.78 ± 0.74 ^b^	0.89 ± 0.28 ^ab^	1.96 ± 1.08 ^a^	1.99 ± 0.60 ^a^
Proteobacteria					
*Desulfovibrionaceae*	0.00 ± 0.00 ^b^	0.03 ± 0.02 ^a^	0.03 ± 0.02 ^a^	0.02 ± 0.01 ^ab^	0.01 ± 0.01 ^ab^
*Parasutterella*	0.00 ± 0.00 ^b^	0.00 ± 0.00 ^b^	0.00 ± 0.01 ^b^	0.00 ± 0.00 ^b^	0.02 ± 0.02 ^a^
Tenericutes					
*Anaeroplasma*	0.65 ± 0.43 ^a^	0.00 ± 0.00 ^b^	0.00 ± 0.00 ^b^	0.00 ± 0.001 ^b^	0.00 ± 0.01 ^b^
*Mollicutes RF39*	0.00 ± 0.00 ^c^	0.00 ± 0.00 ^c^	0.00 ± 0.00 ^c^	0.02 ± 0.01 ^ab^	0.03 ± 0.04 ^a^

^a–c^ Different letters within a row indicate significant differences among treatment groups (*p* < 0.05).

## Data Availability

All data supporting the findings of this study are available within the article and its Supplementary Information. Additional data are available from the corresponding author upon reasonable request.

## Supplementary Materials

The following supporting information can be downloaded at: https://www.mdpi.com/article/10.3390/nu18060925/s1, Table S1: Biochemical traits in mice fed a high-fat diet with α-lipoic acid, betaine, and L-carnitine.

## Abbreviations

The following abbreviations are used in this manuscript:

16S rRNA	16S ribosomal RNA
AL	α-lipoic acid
ASVs	Amplicon sequence variants
BT	Betaine
BW	Body weight
CON	Control diet (10% crude fat)
FDA	Food and Drug Administration
HFD	High-fat diet (60% crude fat)
HFD-AL	High-fat diet supplemented with α-lipoic acid
HFD-BT	High-fat diet supplemented with betaine
HFD-LC	High-fat diet supplemented with L-carnitine
HDL	High-density lipoprotein
KEGG	Kyoto Encyclopedia of Genes and Genomes
LC	L-carnitine
LDA	Linear discriminant analysis
LDL	Low-density lipoprotein
LEfSe	Linear discriminant analysis effect size
OTUs	Operational taxonomic units
PCoA	Principal coordinate analysis
PICRUSt	Phylogenetic Investigation of Communities by Reconstruction of Unobserved States
QIIME 2	Quantitative Insights into Microbial Ecology 2
TC	Total cholesterol
TG	Triglyceride
WHO	World Health Organization
