# Supplementary material for: Effect of Alpha-Lipoic Acid, Betaine, and L-Carnitine Supplementation on Gut Microbiota and Obesity Biomarkers in Mice
Source: Nutrients. 2026 Mar 14;18(6):925. doi: 10.3390/nu18060925 (PMC13029658; doi:10.3390/nu18060925)
Supplement: Supplementary file 1 [file nutrients-18-00925-s001.zip › nutrients-4173907-supplementary.pdf]

Supplementary Information

Table S1

Biochemical traits in mice fed high-fat diet with a-lipoic acid, betaine, L-carnitine

Traits	CON	HFD	HFD-AL	HFD-BT	HFD-LC
Initial body weight	18.04±0.112	18.04±0.154	18.06±0.317	18.06±0.246	18.04±0.374
Final body weight	27.14±0.759	34.60±0.944	30.42±0.558	29.04±0.556	32.28±0.874
Serum leptin	7.14±0.304	27.07±0.402	12.56±0.451	8.96±0.341	17.58±0.503
Serum TG	32.63±1.228	57.67±4.702	49.33±5.239	24.67±4.807	24.33±0.333
Serum TC	139.00±3.606	172.00±0.577	153.67±6.839	141.00±5.568	152.67±1.202
Serum LDL	45.14±5.668	68.80±5.948	56.80±3.863	50.40±0.902	55.47±0.371
Hepatic TG	450.82±28.160	1704.31±53.459	1431.02±146.162	1232.55±117.029	1170.63±131.934
Hepatic TC	124.62±3.201	162.27±1.578	137.29±2.896	131.29±1.233	128.97±2.191
Hepatic HDL	2.92±0.326	1.48±0.365	1.90±0.267	1.50±0.332	1.68±0.270
Size of epididymal adipose tissue	74.97±0.829	106.87±1.481	90.11±0.771	87.87±1.793	90.86±1.249

Means±SE. CON, control group comprising animals fed with a commercial mice chow containing 10% crude fat; HFD, high-fat diet group comprising animals fed with a commercial mice chow containing 60% crude fat; HFD-AL, high-fat diet with a-lipoic acid (AL) group comprising mice fed with 300 mg/kg body weight (BW) of AL; HFD-betaine (BT), high-fat diet with BT group comprising mice fed with 300 mg/kg BW of BT; HFD-LC, high-fat diet with L-carnitine (LC) group comprising mice fed with 300 mg/kg BW of LC. This data was derived from our previous study (Jang et al., 2014).

Reference

[dataset] Jang, A., Kim, D., Sung, K. S., Jung, S., Kim, H. J., & Jo, C. (2014). *The effect of dietary α-lipoic acid, betaine, l-carnitine, and swimming on the obesity of mice induced by a high-fat.* Biochemical traits Data. https://doi.org/10.1039/C4FO00246F
